# Dynamics of the Hypoxia—Induced Tissue Edema in the Rat Barrel Cortex *in vitro*

**DOI:** 10.3389/fncel.2018.00502

**Published:** 2018-12-18

**Authors:** Elvira Juzekaeva, Azat Gainutdinov, Marat Mukhtarov, Roustem Khazipov

**Affiliations:** ^1^Laboratory of Neurobiology, Institute of Fundamental Medicine and Biology, Kazan Federal University, Kazan, Russia; ^2^Aix Marseille Univ, INSERM, INMED, Marseille, France

**Keywords:** brain ischemia, hypoxia, edema, swelling, cortex

## Abstract

Cerebral edema is a major, life threatening complication of ischemic brain damage. Previous studies using brain slices have revealed that cellular swelling and a concomitant increase in tissue transparency starts within minutes of the onset of metabolic insult in association with collective anoxic spreading depolarization (aSD). However, the dynamics of tissue swelling in brain slices under ischemia-like conditions remain elusive. Here, we explored the dynamics of brain tissue swelling induced by oxygen-glucose deprivation (OGD) in submerged rat barrel cortex slices. Video monitoring of the vertical and horizontal position of fluorescent dye-filled neurons and contrast slice surface imaging revealed elevation of the slice surface and a horizontal displacement of the cortical tissue during OGD. The OGD-induced tissue movement was also associated with an expansion of the slice borders. Tissue swelling started several minutes after aSD and continued during reperfusion with normal solution. Thirty minutes after aSD, slice borders had expanded by ~130 μm and the slice surface had moved up to attain a height of ~70 μm above control levels, which corresponded to a volume increase of ~30%. Hyperosmotic sucrose solution partially reduced the OGD-induced slice swelling. Thus, OGD-induced cortical slice tissue swelling in brain slices *in vitro* recapitulates many features of ischemic cerebral edema *in vivo*, its onset is tightly linked to aSD and it develops at a relatively slow pace after aSD. We propose that this model of cerebral edema *in vitro* could be useful for the exploration of the pathophysiological mechanisms underlying ischemic cerebral edema and in the search for an efficient treatment to this devastating condition.

## Introduction

Cerebral edema is a major life threatening complication of ischemic brain damage (Stokum et al., 2016; Zheng et al., 2016). Cerebral edema causes a mass-effect with distortion, tissue shift and increased intracranial pressure leading to cerebral herniation, further brain damage, and death (Frank, 1995; Schwab et al., 1996; Dostovic et al., 2016). However, efficient medical treatment of edema in stroke patients including hyperventilation, hyperosmotic therapy, diuretics, corticosteroids, or barbiturates is limited. Severe cases of edema require surgical decompression to create space to accommodate the increased volume created by the swollen brain by opening the cranial vault and dura, or even by removing non-viable or non-essential brain tissue (Dostovic et al., 2016; Zheng et al., 2016).

Cerebral edema is caused by an increase in the brain tissue water content as a result of osmotic changes caused by ionic gradient disturbances and an accumulation of osmotically active metabolites, such as lactate, in the ischemic region (Stokum et al., 2016). Edema develops through several stages starting with cytotoxic edema, or cellular swelling, manifesting minutes after metabolic insult as a result of water translocation from the extracellular to intracellular compartment, generated by the disturbance in the transmembrane ionic gradients during anoxic spreading depolarization (aSD) (Dreier et al., 2018). Cytotoxic edema starts to develop during aSD and it is characterized by swelling of neurons and glial cells, beading of dendrites, shrinkage of the extracellular space, and changes in optical tissue transparency (Vanharreveld, 1957; Murphy et al., 2008; Risher et al., 2009, 2010, 2012). Similar phenomena are also observed in submerged cortical slices exposed to oxygen-glucose deprivation (OGD) (Obeidat et al., 2000; Andrew et al., 2007; Risher et al., 2009; Brisson and Andrew, 2012).

Despite a wealth of knowledge on the dynamics of cellular swelling, whose onset is tightly linked to aSD, the dynamics of tissue swelling are less well-understood. Importantly, cellular swelling may occur without tissue swelling. If water access to the ischemic tissue is limited, cellular swelling occurs through a translocation of water from the extracellular to intracellular space without any change in the total brain tissue volume. For example, during brain ischemia evoked by cardiac arrest, the total volume of brain tissue, and its water content do not change. Similarly, the ischemic core does not increase in size despite cytotoxic swelling reviewed in Klatzo (1987) and Stokum et al. (2016). However, significant tissue edema may occur if external water access to the ischemic tissue is available, for example, in submerged slice preparations, where the slice is exposed to electrolytes and water in the superfusion medium *ad libitum*. Indeed, water content increases in brain slices exposed to the ischemia-like conditions including OGD and other metabolic insults (Kumar et al., 2006; Elkin et al., 2010). Accordingly, an increase in the slice volume after a combination of cyanide poisoning with aglycaemia could already be detected 30 min after metabolic insult (Elkin et al., 2010). Also, previous studies have shown technical challenges in time-lapse imaging from brain slices caused by shifts of the focal plane during OGD which required sampling of the optical section on the fly, and re-centering and adjusting the field of focus prior to acquiring image stacks (Andrew et al., 2007; Risher et al., 2009). A similar technical challenge was indicated in patch-clamp recordings from the intact hippocampus *in vitro*, where electrical access to the neurons being recorded was often lost after OGD-induced aSD likely because of tissue movement (Dzhala et al., 2001). However, the dynamics of ischemic brain mass tissue movement and edema in brain slices have not been previously addressed.

In the present study, we explored the dynamics of the tissue movement and volumetric changes occurring in slices of the rat barrel cortex during ischemia-like OGD conditions. We found that OGD efficiently induces mass tissue movement and swelling that are characteristic of ischemic cerebral edema, that the onset of tissue swelling is tightly linked to aSD and that it develops on a relatively slow time scale after aSD.

## Materials and Methods

### Ethical Approval

All animal-use protocols followed the guidelines of the French National Institute of Health and Medical Research (INSERM, protocol N007.08.01) and the Kazan Federal University on the use of laboratory animals (ethical approval by the Institutional Animal Care and Use Committee of Kazan State Medical University N9-2013).

### Brain Slices Preparation

Wistar rats (16–23 days old) of either sex were used. Animals were decapitated under isoflurane anesthesia (5%) and the brain was rapidly removed and placed in ice-cold (2–5°C) slicing solution [modified from, (Dugue et al., 2005)] of the following composition (in mM): K-Gluconate 140, Na-Gluconate 15, NaCl 4, EGTA 0.2, D-AP5 50 μM, and HEPES 10 (pH 7.4). Four hundred μm thick thalamocortical slices were cut using a PELCO easiSlicer™ vibratome (Ted Pella, Inc., Redding, CA, USA). Slices containing the barrel cortex were selected by anatomical coordinates (Khazipov et al., 2015) and the presence of barrel structures in L4. Slices were first kept in oxygenated (95% O_2_-5% CO_2_) artificial cerebrospinal fluid (ACSF) of the following composition (in mM): NaCl 126, KCl 3.5, CaCl_2_ 2, MgCl_2_ 1.3, NaHCO_3_ 25, NaH_2_PO_4_ 1.2, and glucose 11 (pH 7.4) for 30 min at 32^o^C and then at room temperature (20–22°C) for at least 1 h before use. For recordings, slices were placed into a submerged chamber and superfused with oxygenated ACSF at 30–32°C at a flow rate of 10 ml/min. Slices were held in place in the recording chamber using a handmade U-shape frame from silver wire (0.025″, A-M Systems, USA). To allow displacement of the tissue during edema, only the two more distant borders of the slice were held by the frame. Oxygen/glucose deprivation (OGD) was induced by superfusion with ACSF in which N_2_ replaced O_2_ and sucrose replaced glucose at equimolar concentration.

### Electrophysiological Recordings

Extracellular recordings of the local field potentials (LFP) were performed in the barrel cortex using single site glass pipette electrodes. Electrodes were pulled from borosilicate glass capillaries (BF150-86-10, Sutter Instrument, Novato, CA, USA) and had resistances of 2–3 MΩ when filled with ACSF. Electrodes were connected via chlorided silver wire to the headstage of a MultiClamp700B patch-clamp amplifier (Axon Instruments, Union City, CA, USA). Recordings were performed in voltage-clamp DC mode, then currents were inverted and voltage calibrated using 5 mV steps.

### Time-Lapse Imaging

Time-lapse microscopy recordings were performed using a BX51WI upright microscope equipped with a dry 4x/0.10 Plan N objective and a water immersed 40x/0.80 LUMPlanFL N objective (Olympus, Tokyo, Japan). The slice was illuminated by a halogen lamp with a 775 nm bandpass filter. Images were acquired using a QIClick-R-F-M-12 CCD camera (QImaging, Surrey, BC, Canada) at 696 × 520 or 1392 × 1040 pixel resolution and 5–10 frames/s acquisition rate.

Time-lapse imaging of the cortical surface with a correction for a change in the surface height was performed either by focusing on the fluorescent dye-filled cells manually or by autofocusing on the highest contrast slice surface images using a home-made Z-correction system. Individual layer 4 neurons located close to the slice surface were labeled with CF™488A fluorescent dye (100 μM) through the patch-pipette in whole-cell mode. The pipette was then removed to enable free cell displacement. The autofocusing system consisted of the step motor, which was attached to the Z-drive of the microscope, and controlled by an Arduino Uno control board, and LabVIEW (National Instruments) software with the Vision Development Module to determine the focal plane of the highest contrast image through online analysis of multiple images taken at different focus distances (range: from +50 to −100 μm, with a 4 μm step). The image was resized to a resolution of 696 × 520 pixels. Then the Sobel operator was used to determine the luminance gradient. The statistical distribution of pixels with different brightness was calculated after the Sobel operator. The average brightness in a given distribution was the criterion for the sharpness of the image. One cycle of autofocusing lasted on average 6.6 ± 0.9 s. Further offline analysis of the position of objects at the cortical surface was used for the detection of tissue drift in the horizontal plane.

### LFP and OIS Analysis

Data were analyzed using custom-written procedures in Matlab (MathWorks, Inc., Natick, MA, USA). OIS was calculated using the first-frame subtraction approach: OIS (t) = (I (t) – I_0_)/I_0_, where I (t)–pixel intensity at the moment t, I_0_-time-averaged pixel intensity in the preconditioned baseline period (100 s). The resulting frames were filtered with a 10 × 10 median filter. Regions of interest (ROIs) were selected as square areas near the recording site. OIS traces were calculated as the average OIS signal within selected ROIs.

LFP signals were downsampled to 1 kHz. The continuous running line fit was removed using local linear regression in 300 s windows with a 10 s overlap [*locdetrend* function from the Chronux toolbox (http://chronux.org/)]. Data were smoothed by the 1,000-point moving average filter and the local negative aSD peak was detected. The baseline level was calculated as the mean value of the LFP in the −20 to −10 s time window preceding aSD. aSD amplitude was calculated as the maximal negative LFP peak from the baseline.

### Statistical Analysis

Statistical analysis was based on the non-parametric Wilcoxon (paired samples) signed rank sum test with the significance level set at *p* < 0.05. Results are given as medians ± interquartile range, where interquartile range is the difference between 75th and 25th percentiles.

## Results

**Figure 1:**
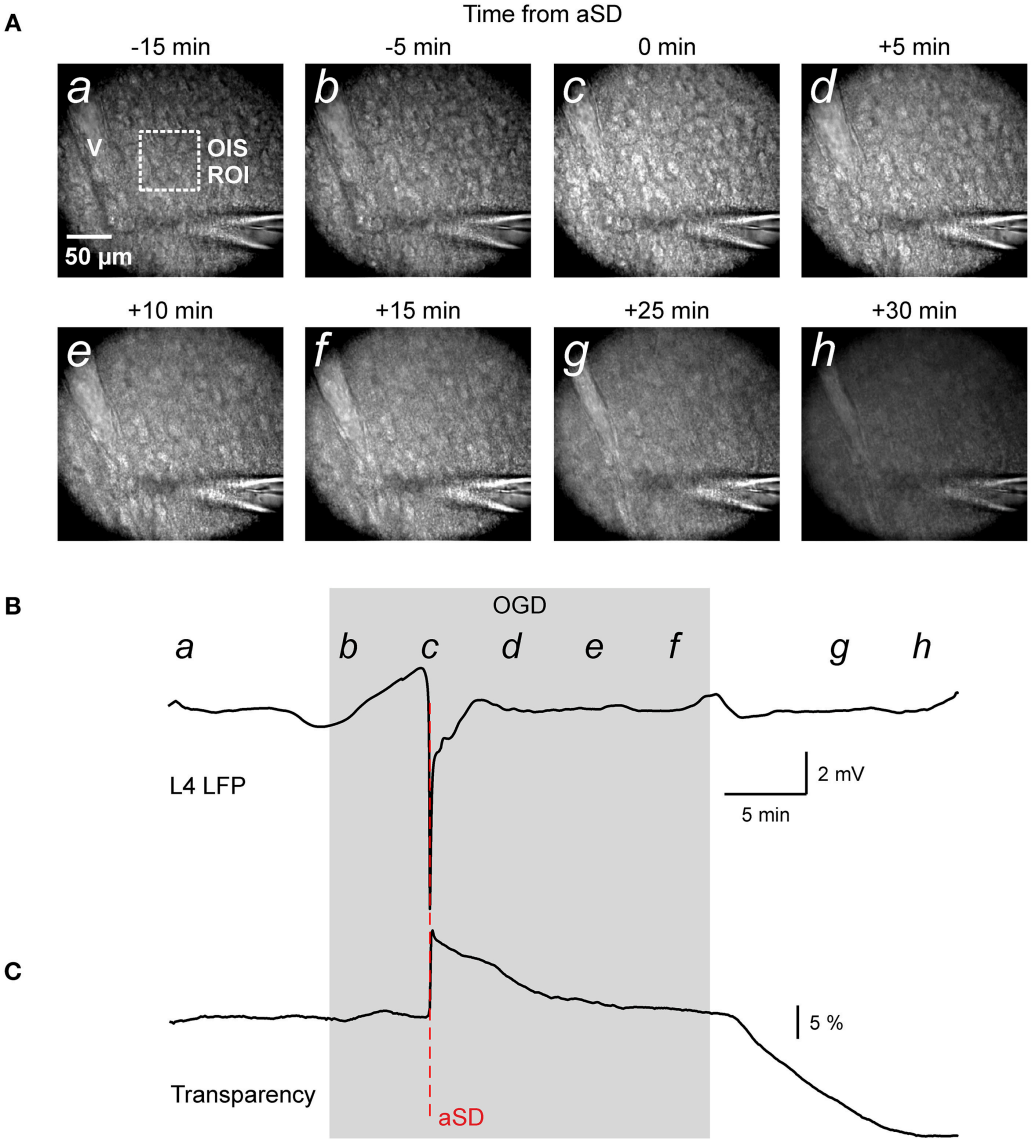
Oxygen-glucose deprivation (OGD) induced mass tissue movement: general observation. **(A)** Snapshots of the cortical slice surface at different time points from the OGD-induced anoxic spreading depolarization (aSD) obtained at constant focal plane set at the slice surface prior to OGD. Note that the tissue starts drifting after aSD and shifts from the focal plane; (a–h) at the top left corner indicate the time points on plot B when the snapshots were taken. Note changes in the vessel (v) appearance due to the vertical tissue displacement. **(B,C)** Corresponding traces of LFP **(B)** and OIS **(C)** recordings from a cortical barrel. Region of interest (ROI) of OIS recording is indicated by dashed box on the snapshot (a). See also corresponding Online Video 1.

We studied the dynamics of the tissue shifts and volumetric changes in slices of the rat barrel cortex during ischemia-like conditions induced by OGD. To this aim, we used time-lapse imaging of the slice surface and borders with concomitant local field potential and OIS recordings. In keeping with the results of a previous study using this preparation (Juzekaeva et al., 2017), OGD induced aSD at a delay of 9.7 ± 3.2 min (*n* = 54 slices) after the onset of perfusion with OGD solution. aSD attained a peak negative value of 5.8 ± 3.9 mV in L4 (*n* = 54) and it was also associated with a sharp transient increase in slice transparency of 25.7 ± 23.2 % dI/I (*n* = 49) (Figures 1B,C; see also summary **Figure 6**).

### Mass Tissue Movement: General Observation

In the initial time-lapse imaging of the cortical surface with a constant focal plane (Figure 1A and Video 1) we observed that during perfusion of slice with OGD solution, the entire cortical tissue started drifting in the horizontal plane as evidenced by the displacement of various objects such as cells and vessels, and vertically as evidenced by the shifting of the objects from the focal plane and the appearance of new moving objects (see also Video 2 at low magnification for a disappearance of vessels). The images also became blurry, losing contrast objects. Particularly impressive was the tissue movement relative to immobile objects such the pipette for extracellular field potential recordings (Video 1). These tissue drifts started in association with aSD, and they continued after aSD (Figures 1B,C). We further attempted to quantify the OGD-induced tissue drifts in several imaging settings.

### Expansion of the Slice Borders

**Figure 2:**
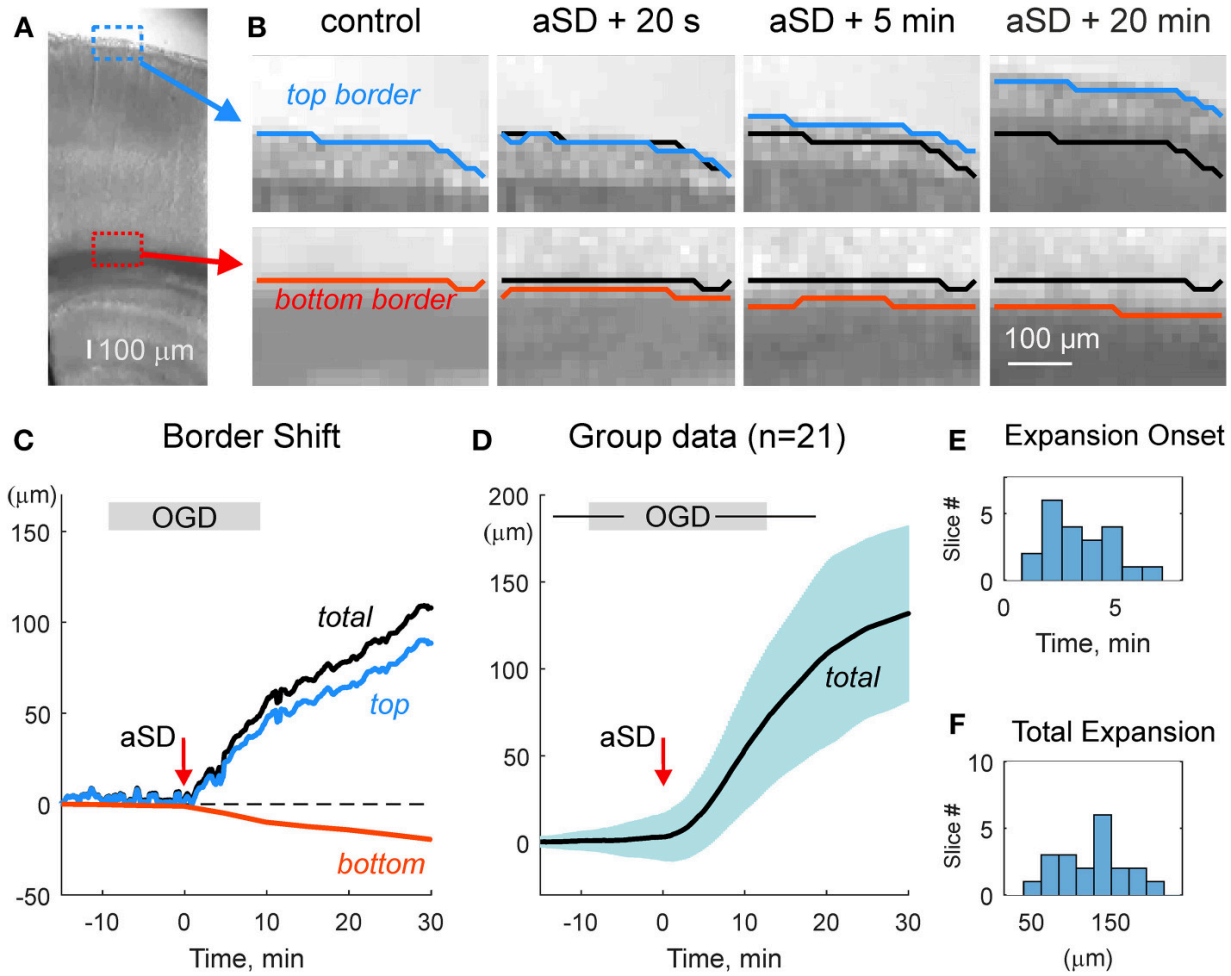
OGD-induced expansion of the cortical slice borders. **(A)** Microphotograph of the cortical slice. ROIs with the meningeal (blue box) and ventricular (red box) borders of the cortical barrel column. **(B)** Snapshots of a position of the top meningeal (blue line) and bottom ventricular (red line) borders of the cortical slice within the ROIs on panel A in control and at different time points from the OGD-induced aSD. Black lines show the pre-OGD borders positions. Note that slice borders start expanding immediately after aSD and continue to expand after reperfusion of the slice with oxygenated glucose-containing ACSF. **(C)** Time course of the OGD-induced top (blue) and bottom (red) borders shift from the control position referenced to aSD as a zero time point in the experiment shown on panels **(A,B)**. Total (black) border shift is a sum of the top and bottom border shifts. Note that shift of the top border is more important than that of the bottom border. **(D)** Average change in the total distance between the top and bottom borders from *n* = 21 slices (shaded area shows SD). Data are referenced to the time aSD = 0. Vertical borders on the gray OGD-box above the plot indicate an average onset and offset of OGD from aSD and horizontal lines indicate SD. **(E,F)** Histograms of the borders expansion onsets **(E)** and total slice border expansion **(F)** 30 min after aSD from *n* = 21 slices. Border movements during the experiment shown on **(A–C)** are also presented in Online Video 2.

We first characterized the shifts of the slice borders. To this aim, we analyzed the position of the top (meningeal) and bottom (ventricular) borders of the cortical slice in the region of somatosensory barrel cortex. As shown on Figures 2A–C and Video 2, slice borders stayed immobile during control conditions, the early phase of OGD, and the OGD-induced aSD. However, immediately after aSD, slice borders started to expand and this process continued after reperfusion of slices with oxygenated, glucose- containing ACSF. Because the onset of the border expansion as well as other volumetric OGD-induced changes (see below) were tightly linked to aSD rather than to the OGD duration, group data analysis was performed in relation to aSD with the aSDs' peak negativity taken as a reference time point T = 0. Borders started to shift from their control position on average 3.5 ± 1.8 min (*n* = 21) after aSD (Figure 2E). Following 29 ± 23 min after aSD (a period that included 15.1 ± 4.5 min in OGD and 8.6 ± 4.1 min of reperfusion), the top (meningeal), and bottom (ventricular) borders expanded by 87 ± 44 and 44 ± 68 μm from their initial pre-OGD positions, respectively (*n* = 21). Corresponding slice width (the distance between the top and bottom borders) increased by 132 ± 76 μm, that is to 107 ± 3 % of the control values (*n* = 21; Figures 2D,F).

### Elevation of the Slice Surface

**Figure 3:**
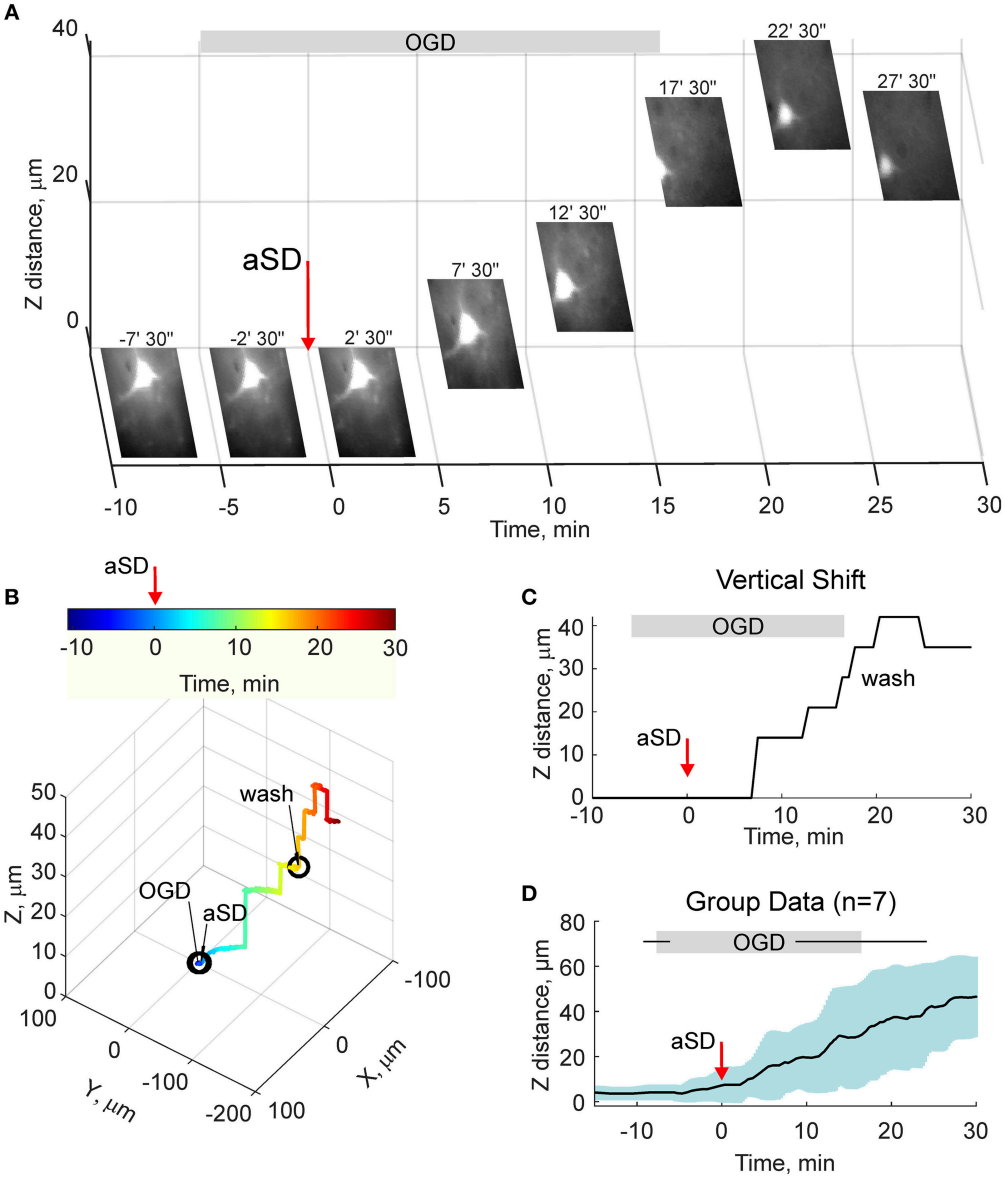
OGD-induced elevation of the cortical slice surface: manual focusing at single cells. **(A)** Snapshots of a CF™488A-labeled L4 neuron located close to the cortical slice surface at different time points from the OGD-induced aSD (exact time points from aSD are indicated above the snapshots). aSD at time = 0 is indicated by vertical arrow. The focal plane was adjusted manually through the time course of the experiment and the corresponding vertical objective displacement is indicated on Z-scale. Note that the cell moves up and horizontally after aSD. **(B)** Corresponding 3D-trajectory of the OGD-induced cell displacement. Time is color-coded. **(C)** Time course of the vertical displacement of a cell is shown on **(A,B)**. **(D)** Group data on the vertical cell displacement in relation to the OGD-induced aSD. Pooled data from *n* = 7 slices.

We next quantified the OGD-induced tissue mass drifts in the middle of the slice at the level of cortical barrels (layer 4). To this aim, we used two approaches. In the first approach, manual focusing on the fluorescent dye-filled cell was used for the tissue displacement estimation. As shown on Figure 3A, the labeled neuron started to drift in the horizontal (x-y) plane shortly after aSD and then disappeared from the focal plane indicating a vertical (z) displacement. Manual correction of the focal plane revealed a displacement of the cell up from the initial position (Figure 3C). The first vertical displacement by 14 ± 3 μm was detected 7.0 ± 1.8 min after aSD (*n* = 7 cells). Continuous manual monitoring of the height of the focal plane of the cell revealed further progressive drift of the cell up and horizontally. In most of the recordings, cells were displaced from the optical field which also required correction of the x-y coordinates of the optical field. Reconstructed cell trajectory in 3D coordinates is shown on Figure 3B (see also Video 3). On average, cells drifted after aSD to attain their final displacement of 42 ± 7 and 36 ± 5 μm (*n* = 7 cells) from the pre-OGD position 30 min after aSD in vertical and horizontal planes, respectively (Figure 3D).

**Figure 4:**
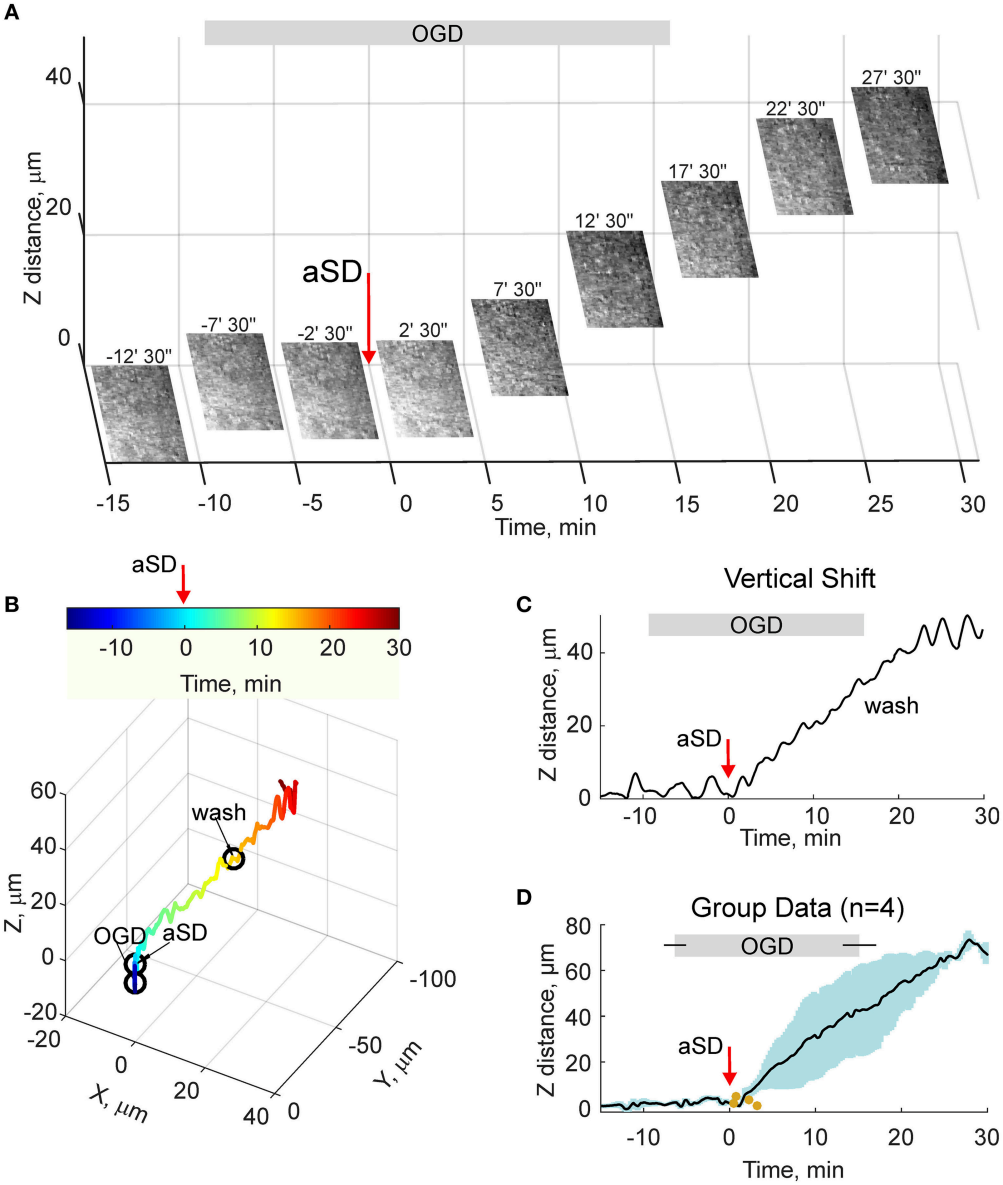
OGD-induced elevation of the cortical slice surface: autofocusing on the slice surface. **(A)** Snapshots of the slice surface at different time points from the OGD-induced aSD (exact time points from aSD are indicated above the snapshots). aSD at time = 0 is indicated by vertical arrow. The focal plane was adjusting automatically during the experiment to the height of the maximal contrast image. Corresponding vertical objective displacement values are indicated on the Z-scale. **(B)** Corresponding 3D-trajectory of the OGD-induced slice surface displacement. Time is color-coded. **(C)** Time course of the vertical displacement of the slice surface. See related Online Video 3. **(D)** Group data on the vertical slice surface displacement in relation to the OGD-induced aSD. Pooled data from *n* = 4 slices. Yellow dots indicate the onsets of the vertical displacement from individual experiments. Layout as on Figure 2D. See also Online Video 3.

Because the estimates of tissue drifts using manual correction of the focal plane are of limited spatial and temporal resolution, we further optimized the detection of the height of the cortical surface using a home-made autofocusing system, which enabled adjustment of the focal plane to the highest contrast image coinciding with the slice surface through online analysis of multiple images taken at different focus distances and feedback to an electric z-drive. Further offline analysis of the position of objects at the cortical surface was used for the detection of tissue drifts in the horizontal plane. Time-lapse imaging with this autofocus system provided results that were similar to those obtained using manual focal plane correction using labeled cell monitoring but at higher spatial and temporal resolution (Figures 4A–C). We found that the tissue starts to drift 1.8 ± 0.8 min (*n* = 4) after aSD to attain a displacement of 71 ± 3 and 26 ± 10 μm (*n* = 4) 30 min after aSD in vertical and horizontal directions, respectively. An elevation of the slice surface corresponded to an increase in the slice thickness to 115 ± 4 % (*n* = 11) of the control value of 400 μm.

### Hyperosmotic Treatment

**Figure 5:**
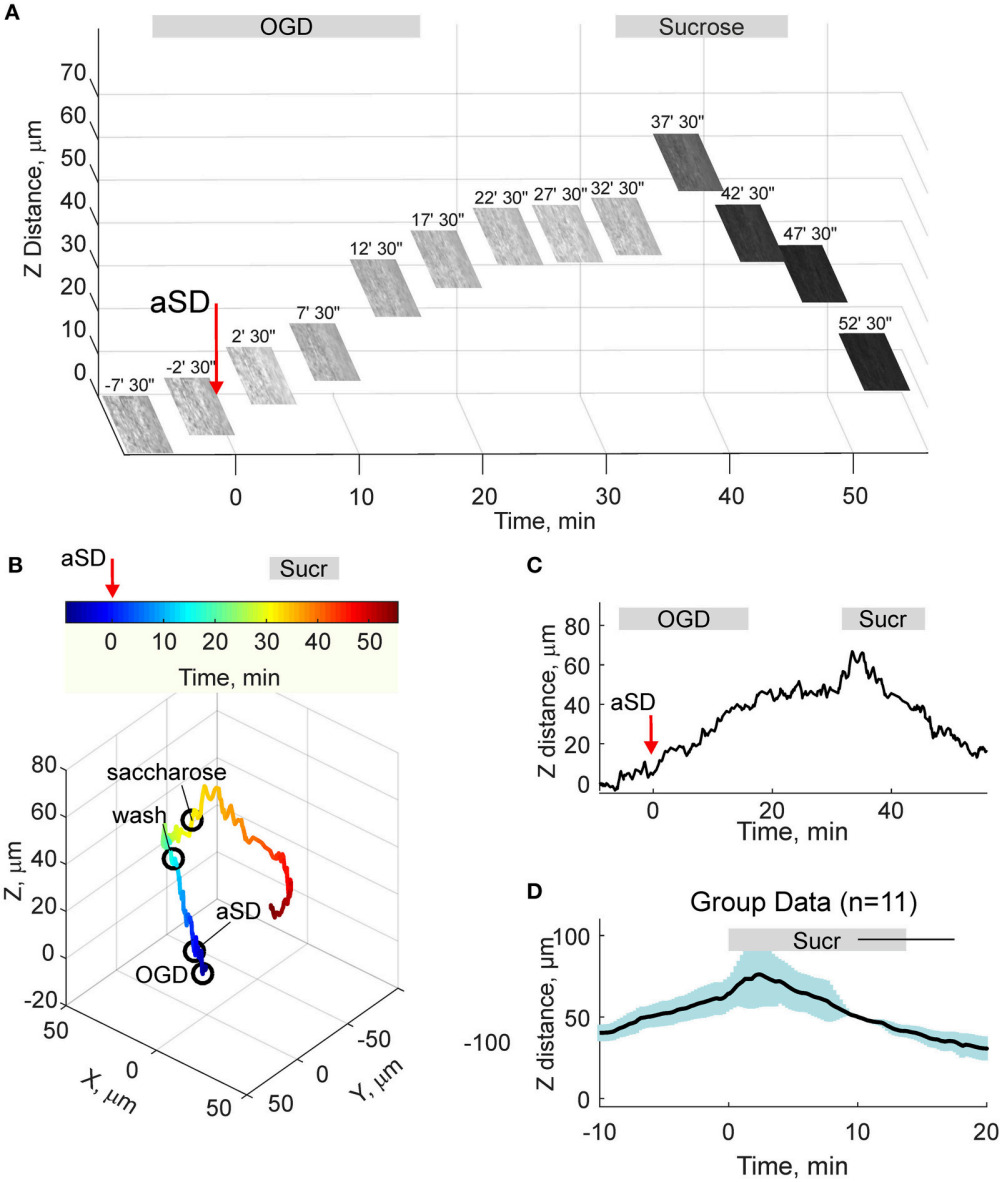
Hyperosmotic treatment reduces OGD-induced slice swelling. **(A)** Snapshots of the slice surface at different time points during experimental osmotic therapy performed after development of the OGD-induced edema. The focal plane was adjusted automatically as on Figure 4. Note that superfusion with hyperosmotic sucrose solution reduces the height of the slice surface. **(B)** Corresponding 3D-trajectory of the slice surface displacement. Time is color-coded. **(C)** Time course of the vertical displacement of the slice surface during OGD and following hyperosmotic treatment. **(D)** Group data on the vertical slice surface displacement in relation to the onset of the hyperosmotic treatment. Pooled data from *n* = 11 slices.

Osmotic therapy is one of the most frequently used clinical treatments for ischemic brain edema. Therefore, we further explored whether osmotic therapy is also efficient in the case of the OGD-induced edema in cortical slices. To this aim, slices which had been previously exposed to OGD and experienced aSD and swelling, were superfused with hyperosmotic ACSF containing 150 mM sucrose and had osmolarity of 472 mOsm compared to 321 mOsm of control ACSF. As shown on Figure 5, hyperosmotic solution evoked a biphasic response with a transient increase in the height of the slice surface attaining a peak of 21 ± 8 μm (*n* = 11) from the pre-sucrose level 2.2 ± 1.0 min after sucrose application, followed by a decrease in the height of the cortical surface to −35 ± 9 μm from the pre-sucrose level 15 min after treatment with hyperosmotic solution. This still remained above the control pre-OGD levels by 35 ± 10 μm (*n* = 11; *p* < 0.05).

### Volumetric Changes

**Figure 6:**
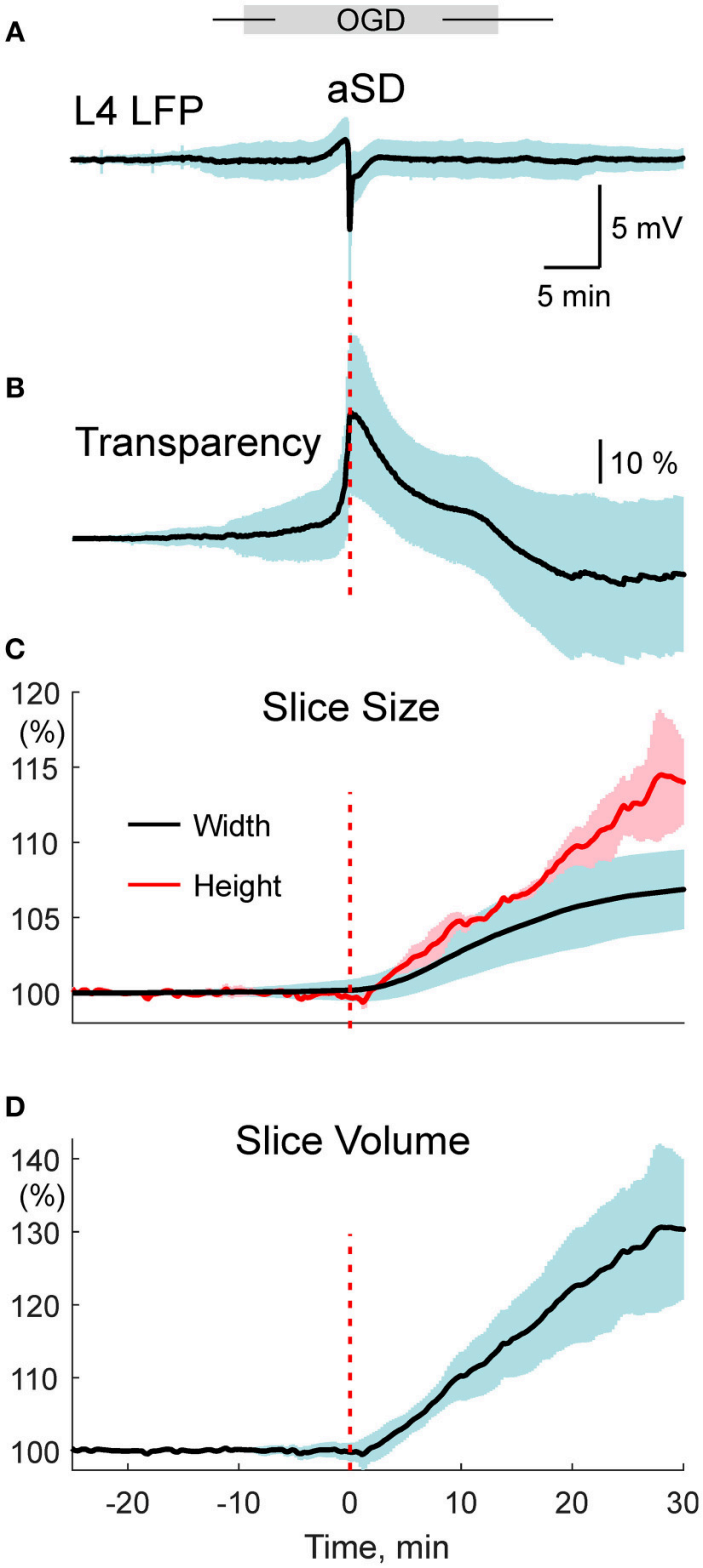
Development of the OGD-induced slice tissue edema in relation to aSD. **(A–D)** Summary plot assembles the time course of the: **(A)** OGD-induced changes in LFP (average of *n* = 54 slices)**, (B)** slice transparency (average of *n* = 49 slices), **(C)** slice size (*n* = 21 slices for the border shifts and *n* = 4 slices for the slice surface shifts with autofocusing method) and **(D)** slice volume estimated from the expansion of the slice borders and elevation of the slice surface. All traces are referenced to electrical aSD.

From the data on the morphometric changes in the slice thickness and border expansion we further attempted to estimate the OGD-induced volumetric changes. We made several assumptions: (i) horizontal expansion is isotropic and therefore a change in the slice surface is proportional to the square of the change in the distance between the slice borders; (ii) vertical shifts of the cortical surface are also isotropic through the entire slice and therefore vertical swelling through the whole slice equals the change at the level of L4. Slice volume changes were calculated according to this formula:

(1) $$\begin{array}{r} \Delta V=\frac{\Delta \;Z\;+\;Z_{0}}{Z_{0}}{\left(\frac{\Delta \;X\;+\;X_{0}}{X_{0}}\right)}^{2} \end{array}$$

where ΔV is slice volume change normalized to the control volume, ΔZ is a change in the slice height, Z_0_ is the initial height, ΔX is a change in the slice width, X_0_ is initial slice width. The results of these calculations are presented on summary Figures 6C,D, were group data on the OGD-induced changes in LFP, slice transparency, slice width (as calculated the from the borders' shifts), and height (from the manual single cell and automatic slice surface shifts pooled together), and slice volume are assembled together, with aSD taken as a reference time point. This plot shows that the slice volume starts increasing almost immediately and linearly after aSD at rate of ~1% per min to attain 30 min after aSD, 130 ± 10 % (estimated from *n* = 21 slices for the border shifts and *n* = 4 slices for the slice surface shifts) of the pre-OGD volume.

## Discussion

In the present study, we characterized the dynamics of the mass tissue movement and volumetric changes occurring in slices of the rat barrel cortex under OGD conditions. Our main findings are that OGD efficiently induces the mass tissue movement and swelling that are characteristic of ischemic cerebral edema, that the onset of tissue swelling is tightly linked to aSD and that the cortical slice edema develops on a relatively slow time scale after aSD.

Our findings on the development of the OGD-induced tissue edema in cortical slices are consistent with the results of previous studies, where an increase in a slice volume by ~20% was observed 30 min after a combination of cyanide poisoning with aglycaemia (Elkin et al., 2010) (present OGD-study: by 30%). This is also in keeping with an increase in the slice water content, which was observed 30 min after the metabolic insult (MacGregor et al., 2003; Kumar et al., 2006; Elkin et al., 2010). It is noteworthy that the final increase in slice volume attained ~75% by 24 h after the onset of metabolic insult (Elkin et al., 2010). Therefore, it is likely that the OGD-induced slice swelling, which was monitored here during the initial stages of the edema formation, also continues after the 30 min interval. The main novelty of the present study consists in showing that the onset of the OGD-induced edema is tightly associated with aSD. Previously, development of cytotoxic edema manifested by swelling of neuronal and glial cell soma, dendritic beading, and shrinkage of the extracellular space was also shown starting minutes after aSD (Vanharreveld, 1957; Murphy et al., 2008; Risher et al., 2009, 2010, 2012). Taking into account these previous findings, our results indicate that the cytotoxic edema and tissue edema in cortical slices develop in parallel, with aSD being their main trigger. We hypothesize that cytotoxic edema is the main driver of the tissue edema in the experimental conditions of submerged brain slice, and that development of cytotoxic edema is supported by continuous and unlimited buffering of the extracellular space by the electrolytes and water contained in the bath medium. This might differ from the *in vivo* settings, where the availability of electrolytes and water in the extracellular space is limited by the lack of blood supply in the ischemic core in the case of focal ischemia or during global brain ischemia caused by cardiac arrest. In the latter cases, cytotoxic edema may develop without prominent tissue edema. However, ionic and water availability through the residual/collateral blood supply, as well as by passive horizontal diffusion may provide substrate for the excessive cytotoxic and tissue swelling that could lead to stronger cellular damage and further aggravate the ischemic state.

Early onset of the tissue swelling after OGD-induced aSD may indicate the particular gravity of this type of metabolic insult in submerged conditions compared to the interface conditions or to the situation *in vivo*. In the submerged slice preparation as used in the present study, the “commitment point” of irreversible damage is achieved during aSD as evidenced by complete and irreversible abolition of synaptic response and membrane potential during OGD episodes with aSD, while shorter OGD episodes without aSD show functional recovery (Rader and Lanthorn, 1989; Tanaka et al., 1997; Joshi and Andrew, 2001; Juzekaeva et al., 2017). Hence, aSD in submerged slices is a terminal event marking neuronal death. In contrast, in brain slices with a gas-fluid interface, where the buffering of the extracellular space is limited (Croning and Haddad, 1998) aSD is not a terminal event and slices can be rescued by reperfusion with oxygen/glucose-containing ACSF several minutes after hypoxia-induced aSD (yet in normal glycemic conditions) (Balestrino and Somjen, 1986; Young et al., 1991; Somjen, 2001). During global ischemia *in vivo*, aSD is also not a terminal event as both morphological and functional impairments to cortical neurons are reversible upon reperfusion started within ~5 min of aSD (Murphy et al., 2008). Moreover, cortical slices prepared up to 5–6 h after cardiac arrest display quite normal physiological and morphological properties (Charpak and Audinat, 1998). Thus, the OGD-induced aSD in submerged slices appears particularly damaging that may be reflected by the early onset of the tissue swelling in association with aSD.

In conclusion, we have shown that cerebral tissue mass movement, distortion, and swelling also occurs under ischemia-like conditions in a cortical slice *in vitro*, that its onset is tightly linked to aSD and that it develops on a relatively slow time after aSD. Evidently, several important elements of ischemic edema are not present in the reduced system of a brain slice *in vitro*, notably vasogenic mechanisms, mechanical constraints that cause an increase in the intracranial pressure, and the swelling component secondary to glymphatic system dysfunction. Therefore, this model is far from fully able to recapitulate ischemic edema *in vivo*. However, this model reproduces cerebral tissue mass movement and the increase in cerebral tissue volume that occur during ischemic brain edema *in vivo* fairly well. Moreover, we found that the OGD-induced slice swelling could be reduced using hyperosmotic treatment, which is one of the most frequently used medical treatments for ischemic brain edema. Therefore, we propose that this model could be useful for the investigation of cellular and molecular mechanisms including specific ionic channels, transporters, and other proteins involved in maladaptive ion and water transport across neuronal and astrocytic membranes during ischemic edema, as well as in the search for more efficient treatment of this devastating condition.

## Data Availability Statement

Original and processed data, and signal processing and analysis routines are available on request from the authors.

## Author Contributions

RK conceived the project. EJ, AG, and MM performed the experiments. AG and EJ analyzed the data. RK wrote the paper.

### Conflict of Interest Statement

The authors declare that the research was conducted in the absence of any commercial or financial relationships that could be construed as a potential conflict of interest.
