# Supplementary material for: Dynamics of the Hypoxia—Induced Tissue Edema in the Rat Barrel Cortex in vitro
Source: Front Cell Neurosci. 2018 Dec 18;12:502. doi: 10.3389/fncel.2018.00502 (PMC6305551; doi:10.3389/fncel.2018.00502)
Supplement: Supplementary file 4 [file Data_Sheet_1.doc]

**Video 1. OGD-induced mass tissue movement** (related to Figure 1). Left, time lapse imaging of a cortical slice in the region of the layer 4. Focal plane is set at the cortical surface and remains constant through the entire recording session. Glass pipette is recording LFP from the middle-depth of the slice. Right plots show the changes in LFP, tissue transparency and a horizontal displacement of the objects 1-3 from their control positions. T=0 corresponds to the peak of anoxic spreading depolarization (aSD). Vertical blue dashed lines on the right plots indicate an onset of OGD and wash of OGD solution with oxygenated, glucose-containing ACSF. Running vertical black line corresponds to the current image displayed on the left. 160× playback speed.

**Video 2.** **OGD-induced slice borders expansion** (related to Figure 2). Left, time lapse imaging of the cortical slice with regions of interest (ROIs) with the meningeal and ventricular borders of the cortical barrel column (white boxes) and interest points of that borders (blue and orange points, respectively). Middle, zoomed (3x) ROIs with initial points positions (black circles) and current border points (blue and orange circles for meningeal and ventricular borders, respectively). Right, LFP (top) and borders shift from the control position (bottom). T=0 corresponds to the peak of aSD. Vertical blue dashed lines on the right plots indicate an onset of OGD and wash of OGD solution with oxygenated, glucose-containing ACSF. Running vertical black line corresponds to the current image displayed on the left. 160× playback speed.

**Video 3. OGD-induced displacement of a CF™488A-labelled L4 neuron located close to the cortical slice surface** (related to Figures 3 and 5). Time lapse images of a fluorescent neuron are shown in 3D coordinates. Focal plane was adjusted manually. 3D cell trajectory with colour-coded time. Inset shows LFP values with a running vertical black line corresponding to the current video frame. Hyperosmotic solution with sucrose was added 31 min after aSD. 160× playback speed.
